# Supplementary material for: Causes and consequences of sex-chromosome turnovers in Diptera
Source: Evol Lett. 2026 Mar 12;10(3):253–68. doi: 10.1093/evlett/qrag003 (PMC13231274; doi:10.1093/evlett/qrag003)
Supplement: qrag003_Supplemental_Files [file qrag003_supplemental_files.zip › Supplementary_Datasets.zip!/Supplementary_Datasets/Dataset_2_Graphical_representation_outgroups/Acroceridae_Acrocera_orbiculus_chromosomes.pdf]

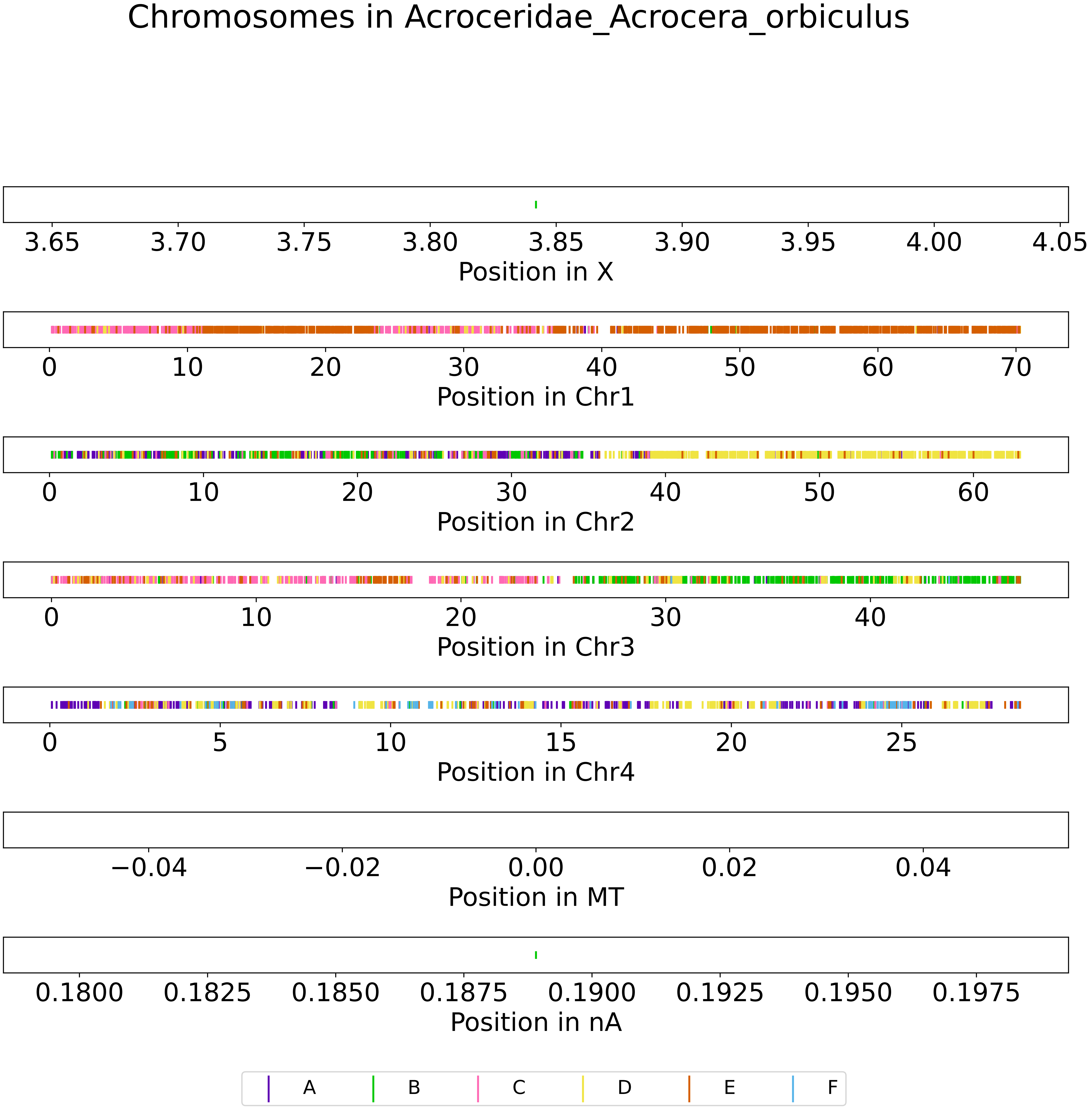

Chromosomes in Acroceridae_Acrocera_orbiculus
3.65
3.70
3.75
3.80
3.85
3.90
3.95
4.00
4.05
Position in X
0
10
20
30
40
50
60
70
Position in Chr1
0
10
20
30
40
50
60
Position in Chr2
0
10
20
30
40
Position in Chr3
0
5
10
15
20
25
Position in Chr4
−0.04
−0.02
0.00
0.02
0.04
Position in MT
0.1800
0.1825
0.1850
0.1875
0.1900
0.1925
0.1950
0.1975
Position in nA
A
B
C
D
E
F
